# Nanoplastics formed during the mechanical breakdown of daily-use polystyrene products†

**DOI:** 10.1039/c8na00210j

**Published:** 2018-12-04

**Authors:** Mikael T. Ekvall, Martin Lundqvist, Egle Kelpsiene, Eimantas Šileikis, Stefán B. Gunnarsson, Tommy Cedervall

**Affiliations:** NanoLund, Lund University Box 118 22100 Lund Sweden; Department of Biochemistry and Structural Biology, Lund University Box 124 22100 Lund Sweden Martin.Lundqvist@biochemistry.lu.se

## Abstract

Large amounts of plastics are released into the environment every day. These released plastics have a clearly documented negative effect on wildlife. Much research attention has been given to large plastic pieces and microplastics. However, if the breakdown of plastics is a continous process, eventually nanoplastics will be produced. Nanoplastics will affect wildlife differently from larger plastic pieces. We have studied the products formed by the mechanical breakdown of two commonly used polystyrene products, takeaway coffee cup lids and expanded polystyrene foam. After breakdown using a food processor, we characterized the breakdown products using seven different methods and found nanosized polystyrene particles with different shapes and negative or nearly neutral surface charges. These results clearly demonstrate that daily-use polystyrene products can break down into nanoparticles. Model polystyrene particles with different sizes and surface modifications have previously been shown to have different negative effects on wildlife. This indicates that breakdown nanoparticles might have the potential to cause cocktail effects in nature.

## Abstract 2

Limited mechanical force produces plastic nanoparticles from commonly used plastic objects.

## Graphical abstract

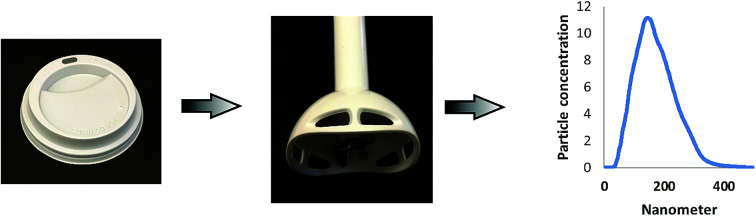

## Introduction

In the last three decades, reports concerning plastics in natural waters have continuously increased, with further intensification since 2010. Recently, 1.15–2.41 million tonnes of plastic were estimated to reach the oceans from rivers and lakes every year,^1^ and the estimated amount of plastics produced globally to 2017 was 8300 million tonnes.^2^ There are many images available showing plastics trapping or filling the gut of animals. Furthermore, there is growing concern that smaller plastic pieces, invisible to the naked eye, might be an even more severe threat to wildlife. Plastic pieces with sizes equal to or smaller than 5 mm are referred to as microplastics. Owing to their small size, microplastics can be trapped by filter-feeding organisms, such as zooplankton and bivalves, and can therefore enter food chain at an early stage. Primary microplastics are industrially manufactured and can be accidentally released into nature during handling. However, most microplastics found in natural waters are breakdown products of larger plastics. The potential sources for new microplastics are large and continuously growing owing to widespread plastic use on a global scale. Since 2015, 6300 million tonnes of plastics had become waste, of which 79% had ended up in landfills or nature.^2^ Today, microplastics are found in many aquatic organisms,^3,4^ which has been suggested to be a future potential global conservation issue for biodiversity.^5^ Recently, microplastics have been found in table salt,^6,7^ commercially cultured mussels,^8,9^ fish,^10–12^ beer,^13^ bottled water,^14^ and tap water.^13^

A subfraction of microplastics with sizes equal to or less than 100 nm in at least one dimension are described as nanoplastics. The direct release of nanoplastics into nature is probably very small and whether or not the breakdown of microplastics continues down to nanosized fragments is debated. It is difficult to detect and identify nanosized plastics in the environment. However, nanoplastics have recently been collected in the North Atlantic,^15^ while styrene monomers and oligomers have been found in sand and water from shorelines worldwide.^16–18^ These styrene monomers and oligomers are most likely breakdown products from polystyrene released into the environment. Furthermore, polystyrene from coffee cup lids and collected weathered plastics has been shown to break down into nanosized particles after being exposed to ultra-violet (UV) radiation in weathering chambers.^1,19^ Polystyrene is among the most extensively used plastics and is found in products including styrene foams, plastic cutlery and glasses, and a large number of packaging products.

Nanoplastics may pose a more severe threat to aquatic wildlife than microplastics owing to the impact of their small size on their environmental fate,^20^ which allows for other exposure scenarios and the invocation of other biological effects.^3,21^ Size-dependent toxicity has been shown in zooplankton.^22,23^ Furthermore, size-dependent uptake has been described in zooplankton,^24^ fish eggs,^25^ fish embryos,^26,27^ and fish,^23,25^ resulting in size-dependent effects on the organisms.^23,28,29^ These uptake- and nanosize-dependent effects allow for complex outcomes. For example, nanoplastics can travel through food webs from algae to zooplankton to planktivorous fish to piscivorous fish.^21,23,30–32^ Furthermore, after food-chain transport, nanoplastics can still affect fish metabolism and behaviour.^21,23,30,32^

In the environment, and after uptake into plants and animals, nanoplastic surfaces will interact with surrounding inorganic and organic matter. This will lead to agglomeration and different surface chemistries that will influence exposure scenarios and secondary interactions with biological systems, and, therefore, produce biological effects. Furthermore, breakdown products under natural conditions are thought to be heterologous in size, morphology, and surface chemistry,^33,34^ which will affect interactions with biological matter. To our knowledge, all studies of the biological effect of nanoplastics to date have been performed using commercially manufactured primary nanoplastics. These are well defined in terms of size and surface chemistry, and are available in reasonable amounts. Studies using manufactured nanoplastics are necessary and will continuously be needed in the future, because they allow for mechanistic studies determining the importance of size, different surface chemistry, and controlled alteration of the particles, which will provide important information about subfraction differences and mechanisms behind these effects. However, it is also important to characterize nanoplastics in nature and determine their impact on biology.^33^

Plastic can be degraded in many ways. Macroplastics can, in the lab, be divided into microplastic by cutting. However, plastics waste that ends up in nature will generally experience other forces. For example, plastic waste that ends up in oceans will be subjected to UV radiation from the sun. Lambert and Wagner have shown in a controlled experiment that three weeks of UV radiation generates plastics particles in the nanometer-size range.^1^ Plastics in the ocean will also experience a complex chemical environment with ions, organic molecules, and different lifeforms that may, in combination with other factors, effect the plastic breakdown. Plastics will also be subjected to mechanical forces, such as friction due to water movement, and wearing against other plastic waste or against sand and rocks at a shore. However, it has not yet been documented whether plastics subjected to different mechanical forces break down into nanosized fragments. To produce larger amounts of mechanically broken-down plastics, we subjected commonly used polystyrene coffee cup takeaway lids and styrene insulation foam to a household blender. After blending, small particles were separated from large debris by filtration and analysed for size and surface chemistry. We found that both materials broke down into nanosized styrene particles. The formed nanoplastics had a surprisingly narrow size distribution, disparate shapes, and a negative or close to neutral surface charge. These results are an important step toward the detection and determination of plastic material breakdown to the nanometer size range in nature.

## Experimental

### Preparation of nanoparticles

The polystyrene plastics used were expanded polystyrene foam board intended for insulation, and ordinary coffee cup lids purchased from a Swedish food chain. For nanoparticle preparation, 2 g of plastic was broken into smaller pieces and placed in a beaker containing 115 mL of milli-Q water. An immersion blender (Bosch ErgoMixx 600W (E-nr: MSM66020/1), Robert Bosch Hausgeräte GmbH, Germany) was used to mix the plastic and water for 5 min at maximum mixing speed. A 20 mL syringe (BD Plastipak) was used to withdraw the solution from the beaker, which was then filtered through syringe filters (Whatman) into a small beaker or flask. Fig. S1 and S2† show this procedure. The 0.45 μm sample was first filtered through a 1.2 μm filter before filtering through a 0.45 μm filter. The coffee cup lid samples were filtered through a 0.8 μm filter after blending. A blank control was prepared by mixing only water and filtering it according to the same procedure. As a polystyrene reference material for the chemical description, we used polystyrene nanoparticles with both positively (amine, PS-NH_2_) and negatively (carboxylated PS-COOH) charged surfaces, obtained from Bang Laboratories Inc., USA.

### Characterization of nanoparticles

#### Chemical description

Attenuated total reflectance Fourier-transform infrared spectroscopy (ATR-FTIR) was performed using a Spectrum One FT-IR spectrometer (Perkin Elmer) equipped with a Universal ATR accessory. The samples were prepared by adding 10 μL of solution to the ATR crystal and allowing it to evaporate. A further 10 μL of the solution was then added, a cap was placed over the sample, and the spectrum scanned. Each spectrum was an average of 50 scans between 4000 and 650 cm^−1^, at a resolution of 4 cm^−1^. The water background signal has been subtracted from each spectrum. To obtain a measurable concentration of the coffee cup lid particles using FT-IR, we concentrated the sample by lyophilizing and resuspending it in a 10-fold smaller volume of milli-Q water. The instrument crystal was cleaned thoroughly between measurements with detergent, ethanol, and water.

Analytical HPLC was performed on an Agilent 1100 series system with a YMC C18 (YMC-Pack ODS-AQ; 250 × 4.6 mm i.d.) column and a 10 mm precolumn of the same material, at a flow rate of 1 mL min^−1^. The mobile phase was water–acetonitrile (25 : 75, v/v), mixed in the system. The method used was based on previously published analyses of polystyrene particles.^35^

Absorbance spectra between 200 and 700 nm were recorded on a Probedrum spectrophotometer (Probation Labs Sweden AB). The sample volume was at least 0.5 mL and each spectrum was an average of 50 scans of the same sample.

#### Morphology and size characterization

The particle samples were examined by transmission electron microscopy (TEM) using a JEOL JEM-1400 PLUS microscope at 100 kV (JEOL Ltd., Japan). Samples were prepared for TEM imaging by pipetting 10 μL of the particle suspension onto a pioloform-coated single slot grid (Ted Pella, Cu, Pelco Slot Grids, USA), after which the water was allowed to evaporate, leaving the particles on the grid. Micrographs were recorded with a JEOL Matataki CMOS camera using TEM Centre for JEM1400 Plus software. The particle size distribution was later analysed from the images using ImageJ image processing and analysis software.^36^

Nanoparticle tracking analysis (NTA) was performed using a NanoSight LM10 instrument (Nanosight, England) equipped with a 405 nm laser. A sample volume of 400 μL was used and 60 s videos were recorded and analysed using NTA 2.3 software (Nanosight, England). Size data are reported as the mean (by concentration) and mode. Data were collected from four independently mixed samples per treatment.

Dynamic light scattering (DLS) measurements were conducted on a DynaPro Platereader-II, Wyatt Technology Corp. USA. The sample volume was 100 μL and each sample was recorded 10 consecutive times with an acquisition time of 10 s at 25 °C. Data were analysed using analysis software Dynamics V7.

#### Surface charge

Zeta potential measurements were performed in water at 25 °C using a Zetasizer Nano ZS instrument (Malvern Instruments, Worcestershire, UK). Measurements were repeated three times and averaged for three consecutive analyses of the same sample.

## Results and discussion

### Preparation of polystyrene nanoparticles

Two different polystyrene products commonly used on a daily basis, namely, expanded polystyrene insulation foam and coffee cup lids, were used as the starting materials for nanoparticle preparation. The breakdown procedure is described in Fig. S1 and S2.† In short, the plastics and water were mixed for 5 min using an immersion blender. Afterwards, large pieces of plastic remained in the beaker, regardless of the starting material. The suspension was removed with a syringe and contained various amounts of small yet visible plastic pieces. However, after filtration through a 1.2 μm syringe filter, the water was clear with no visible pieces or cloudiness. The preparation was stored at 23 or 4 °C without any visible aggregation for at least two weeks.

We used seven techniques to verify the presence of polystyrene and characterize the polystyrene particles in the prepared samples, and determine its size distribution, shape, and surface charge, as shown in Table 1.

**Table 1:** Methodology used to determine chemical and morphological characteristics of the produced nanoparticles

Chemical composition	Shape	Size distribution	Surface charge
ATR-FTIR	TEM	NTA	Zeta-potential
Absorbance		DLS	Analytical HPLC
Analytical HPLC		TEM	

As material was possibly released from the blender itself during mixing, control experiments were performed using only water and analysed by NTA. In our experience, NTA is the most sensitive method of the seven for detecting particles. Only a few particles from the blender were detected in the control sample, as shown in Fig. S6.† However, a new blender from a different brand generated a significant amount of particles in the control sample (see text in ESI†).

### Characterization of chemical composition of produced material

**Fig. 1:**
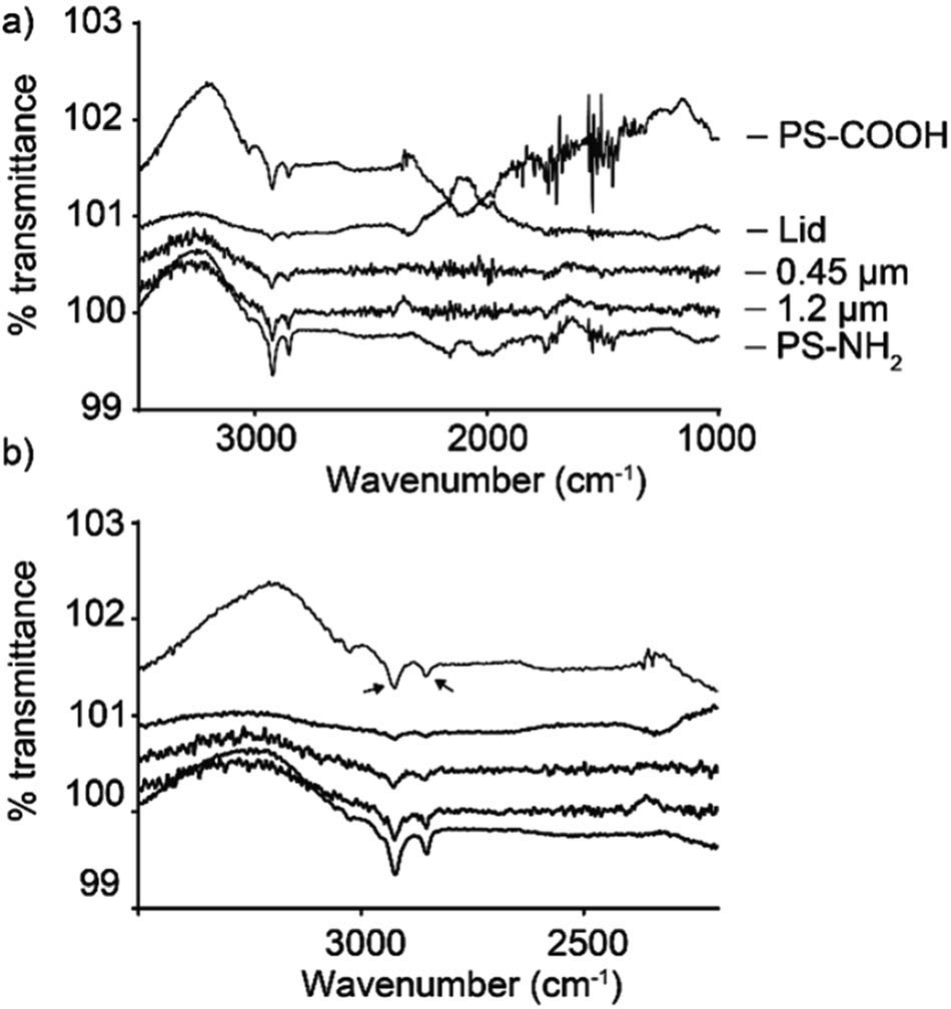
Chemical composition characterized by ATR-FTIR. Lid is the sample prepared from coffee cup lids, 0.45 μm is the expanded foam sample filtered through a 0.45 μm syringe filter, 1.2 μm is the expanded foam sample filtered through a 1.2 μm syringe filter. (a) Shows the spectrum in the wavenumber range 1000–3500 cm^−1^; (b) shows the same spectrum in the wavenumber range 2200–3500 to more easily observe the bands at 2925 and 2854 cm^−1^, as indicated by arrows in (b).

ATR-FTIR was used to determine whether the produced materials contained polystyrene. Fig. 1a shows results obtained for samples from the coffee cup lids and expanded foam. The prepared samples had clear similarities with the PS-COOH and PS-NH_2_ control samples. ATR-FTIR is an increasingly applied technique in particle analysis.^37–39^ Owing to the low concentration, some absorbance peaks are lost, but clear C–H stretches were observed at 2925 and 2854 cm^−1^, in addition to the aromatic C=C stretch at 1542 cm^−1^.

**Fig. 2:**
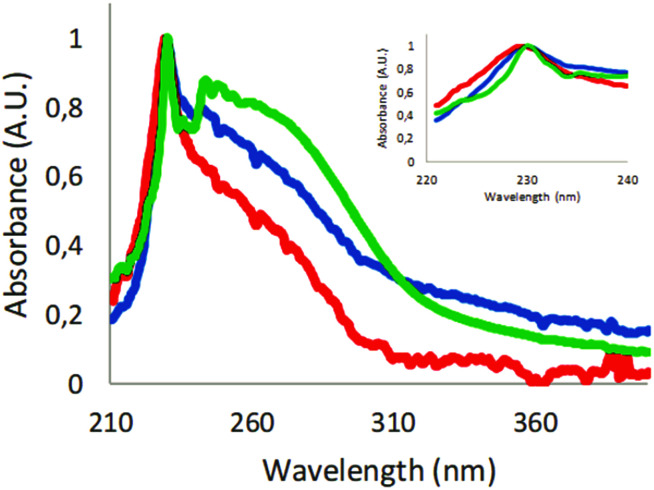
Absorbance scans. Green: concentrated coffee cup lid particles; blue: expanded polystyrene foam filtered through 1.2 μm filter; red: expanded polystyrene sample filtered through an additional 0.45 μm filter. Inset: magnified polystyrene peak at around 230 nm. The absorbance scans were normalized against the highest value at around 230 nm in each sample.

The absorbance of polystyrene nanoparticles will be influenced by the polymeric chain and scattering from larger particles. The absorbance scan of 60 nm carboxyl-modified polystyrene particles showed an absorbance peak at around 230 nm (Fig. S3†). Absorbance scans of samples generated from expanded polystyrene foam had the same characteristic peak at around 230 nm. In the sample from the coffee cup lid, we could not detect any absorbance, probably owing to the concentration being too low. Therefore, the coffee cup lid sample was concentrated by lyophilizing and resuspending in a 10-fold smaller volume. As shown in Fig. 2, the absorbance spectra from the resuspended sample were similar to the foam spectra. In addition to the peak at 230 nm, there was a broad shoulder at longer wavelengths, indicating additional materials in the samples.

Lyophilisation and resuspension caused partial aggregation of the particles (Fig. S6†). The absorbance spectra of samples from expanded polystyrene foam and coffee cup lids clearly showed that polystyrene was present in our samples.

**Fig. 3:**
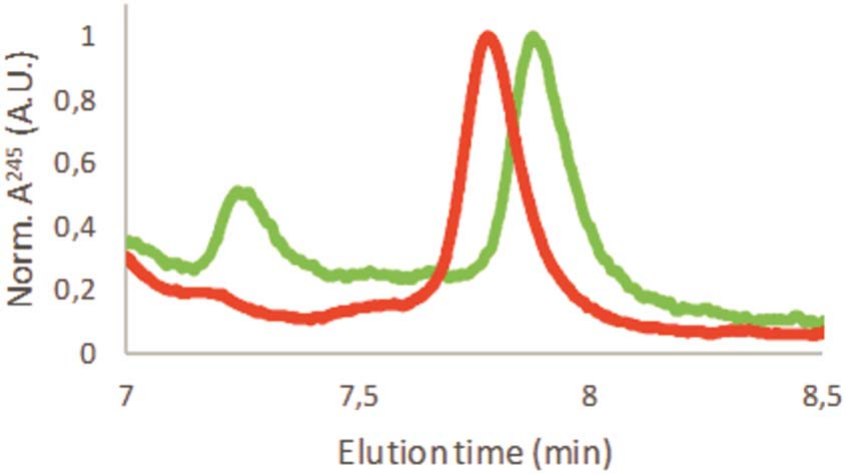
Analytical HPLC results. Coffee lid sample in green and expanded foam sample in red. Data shown are normalized against the polystyrene elution peak.

Polystyrene can be separated using analytical HPLC.^35^ The results shown in Fig. S4† using commercially available polystyrene particles indicated that HPLC can separate the particles according to their surface charge but not size. However, these findings require further investigation and are beyond the scope of this article. Fig. 3 shows elution spectra for the coffee cup lid sample (in green) and the expanded foam samples (in red). The scans are zoomed in at the polystyrene elution time, determined using commercially available polystyrene particles, as shown in Fig. S4.† Both samples were filtered through a 0.2 μm filter before loading on the HPLC column to prevent large debris entering the columns. The samples showed at least one peak in the region where polystyrene was expected to be eluted. The reproducibility of HPLC was shown in Fig. S5,† in which three repeats of the expanded foam sample are shown. Finally, one additional species was eluted later than the prepared plastic by HPLC, indicating that a material other than polystyrene was present in the samples. For example, Fig. S6† shows the whole elution spectrum for a commercially available polystyrene particle (PS-COOH) and an expanded foam sample. This figure shows that the PS-COOH sample is significantly purer than our produced particles. The broad elution profile between 2 and 6 min for the expanded foam sample might be a reason for the broad absorbance peak observed in Fig. 2.

### Shape of formed polystyrene material

**Fig. 4:**
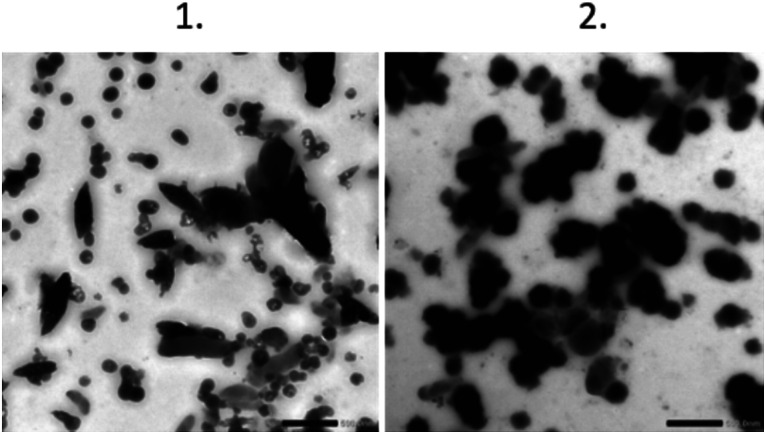
TEM pictures of the particles. Scale bar is 0.5 μm. Panel 1 is the 1.2 μm filtered sample and panel 2 is the 0.45 μm filtered sample.

The shapes of the produced polystyrene particles were characterized using TEM. For the expanded foam sample, TEM images of the particles were obtained, as shown in Fig. 4. Interestingly, many different shapes were present after filtration through the 1.2 μm filter. The nonspherical shapes seemed to be larger and disappeared when the pore size of the filters decreased. Particles smaller than 100 nm were clearly present after all filtration steps, with the size of the particle mix decreasing with each filtration step. We were not able to obtain TEM images of the coffee cup lid sample, possibly due to the low concentration.

### Size of prepared material

Two distinct particle shapes were observed in the TEM analysis of the 1.2 μm filtered material, namely, spherical particles with a mean diameter of approximately 125 nm, and more elongated particles with an approximate mean length and width of 437 nm and 173 nm, respectively (Table 2, Fig. 4(1)). The sample filtered through the 0.45 μm filter (Fig. 4(2)) contained particles of a spherical shape with a mean diameter of approximately 201 nm (Table 2). However, the particles in the 0.45 μm filtrate started to form larger aggregates.

**Table 2:** Size characterization of produced particles; values represent means ± standard deviation

	Coffee cup lid	0.45 μm filter	1.2 μm filter
NTA mode (nm)	104 ± 6	135 ± 12	155 ± 9
NTA mean (nm)	125 ± 67	154 ± 52	182 ± 69
DLS (nm)		167 ± 4	227 ± 5
*Polydispersity*		% Pd 24	Multimodal
TEM (nm)			
*Spherical*		*Ø* 201 ± 45	*Ø* 125 ± 24
*Elongated*			*L* = 437 ± 120
			*W* = 173 ± 26
*ζ*-Potential (mV)	−7.1 ± 44	−14.3 ± 32	−36.1 ± 6

**Fig. 5:**
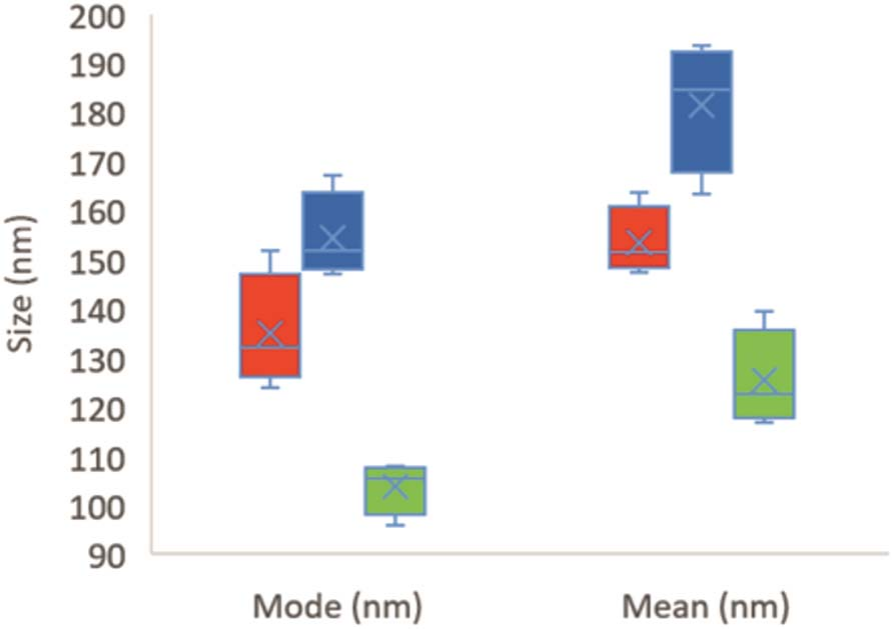
Boxplot of NTA results. Red is expanded foam sample filtered through 1.2 μm and then 0.45 μm filter; blue is expanded foam sample filtered through 1.2 μm filter; green is the coffee cup lid sample. Both the mode and mean size are shown. Whiskers show maximum and minimum values, crosses show mean values and horizontal lines show median values. Each treatment is based on the measurement of four individual samples.

The NTA results from four individual experiments (four samples mixed with the blender) for each sample are shown in Fig. 5 and summarized in Table 2. Examples of raw data are shown in Fig. S6.† The size distributions were surprisingly narrow (Fig. S6†) considering the production method used.

One striking observation from the NTA results for the expanded foam samples was that, even when a 1.2 μm filter was used, no particles larger than 700 nm were detected, which is also in line with the TEM results. The coffee cup lid generated particles of smaller size than the expanded foam. No major shift in the size distribution was observed for the coffee cup lid sample when filtered through filters of different sizes. Lyophilization and resuspension of the coffee cup lid particles increased the particle concentration, but also resulted in larger particle sizes and a broader size distribution, as shown in Fig. S6.† However, as this result was due to the lyophilization process, we did not refilter the suspension through differently sized filters. Table 2 shows the different particle sizes detected in the samples with NTA. We also measured the produced samples using DLS. However, owing to the particle concentration and, for DLS, broad particle size range, it was difficult to obtain reliable data (see Fig. S7 and S8,† and Table 2).

### Surface chemistry

**Fig. 6:**
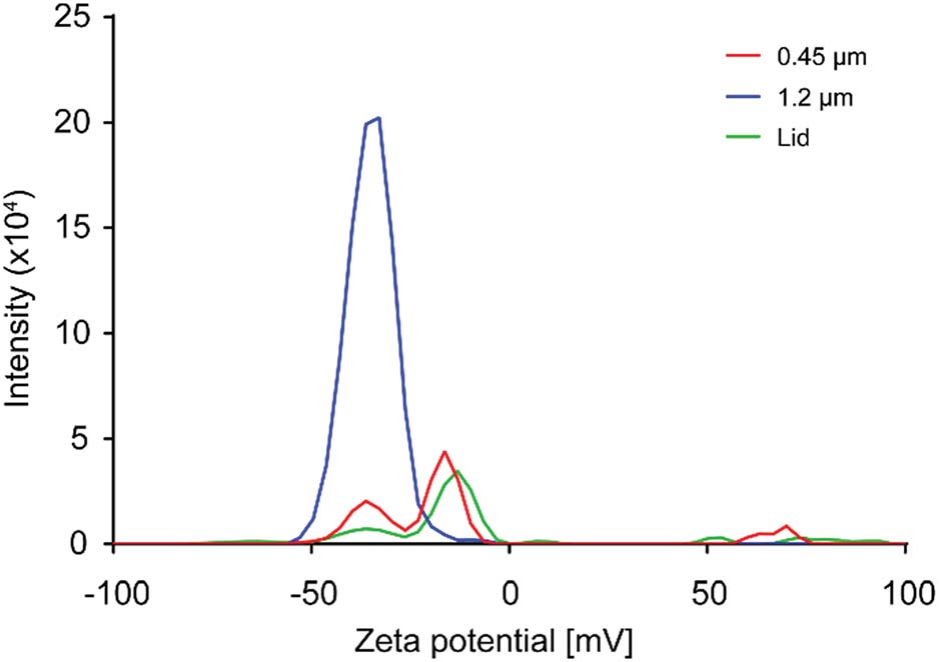
Results from zeta potential measurements. Coffee cup lid sample in green, expanded foam sample filtered through a 1.2 μm filter in blue, and expanded foam sample filtered through a 0.45 μm filter in red.

The surface charge of the produced material was characterized by measuring the zeta-potential. As the particle concentrations in the prepared samples were too low to be optimal for zeta potential measurements, and resulting values should only be taken as an indication of the sample zeta potential. In Fig. 6, the result for the coffee cup lid sample is shown in red. The sample showed a broad distribution of charges, with the most prevalent peak at −12 mV. However, more and less negatively charged species were present, indicating particles with different surface chemistry. The expanded foam sample filtered through a 1.2 μm filter, shown in blue in Fig. 6, gave a reliable zeta-potential reading of −36 mV, while the sample filtered through a 0.45 μm filter, shown in red, gave a broad distribution of charges that resembled the results from the coffee cup lid, with the most prevalent peak at −14 mV. This indicated that larger particles were more negatively charged than smaller particles. This might be due to larger particles with nearly neutral surface charges quickly aggregating, while smaller particles remain in the dispersion longer.

## Conclusions

This work aimed to crudely mimic the natural breakdown process originating from mechanical force, such as garbage repeatedly being dragged over sand and stones by waves on a shoreline, to determine whether that process could lead to the breakdown of products to the nanoscale. We showed that just 5 min of mechanical treatment successfully generated nanoparticles. The breakdown polystyrene nanoparticles from coffee cup lids and expanded polystyrene foam showed surprisingly narrow size distributions. The shape of the formed nanoparticles was not only spherical, but also elongated. Finally, the surface charge varied from negative for larger particles to almost neutral for smaller particles. Together, these results highlight the potential release of nanosized plastic particles into nature by exposure to mechanical force, a process that occurs along coastlines worldwide, where plastic materials are continuously moved by wind and waves, and scraped over rocks, sand, and other hard materials. The small but varying sizes, different shapes, and broad surface charge distributions of these particles indicate that mechanical breakdown can be expected to produce plastic nanoparticles resembling all the different kinds of model particles usually used in ecological and toxicological nanoplastic studies. This is problematic because different sizes, shapes, and surface charges have been shown to negatively affect organisms in different ways, leading to a possible cocktail effect of nanoplastics in nature. These results are a first step toward the detection and characterization of the breakdown of plastics to materials in the nanometer size range in nature.

## Conflicts of interest

There are no conflicts to declare.

## Supplementary Material

NA-001-C8NA00210J-s001
