# Supplementary material for: Nanoplastics formed during the mechanical breakdown of daily-use polystyrene products
Source: Nanoscale Adv. 2018 Dec 4;1(3):1055–61. doi: 10.1039/c8na00210j (PMC9473236; doi:10.1039/c8na00210j)
Supplement: NA-001-C8NA00210J-s001 [file NA-001-C8NA00210J-s001.pdf]

Electronic Supplementary Material (ESI) for Nanoscale Advances.
This journal is © The Royal Society of Chemistry 2018

Supplementary information

Procedure to make polystyrene nanoparticles from large polystyrene pieces

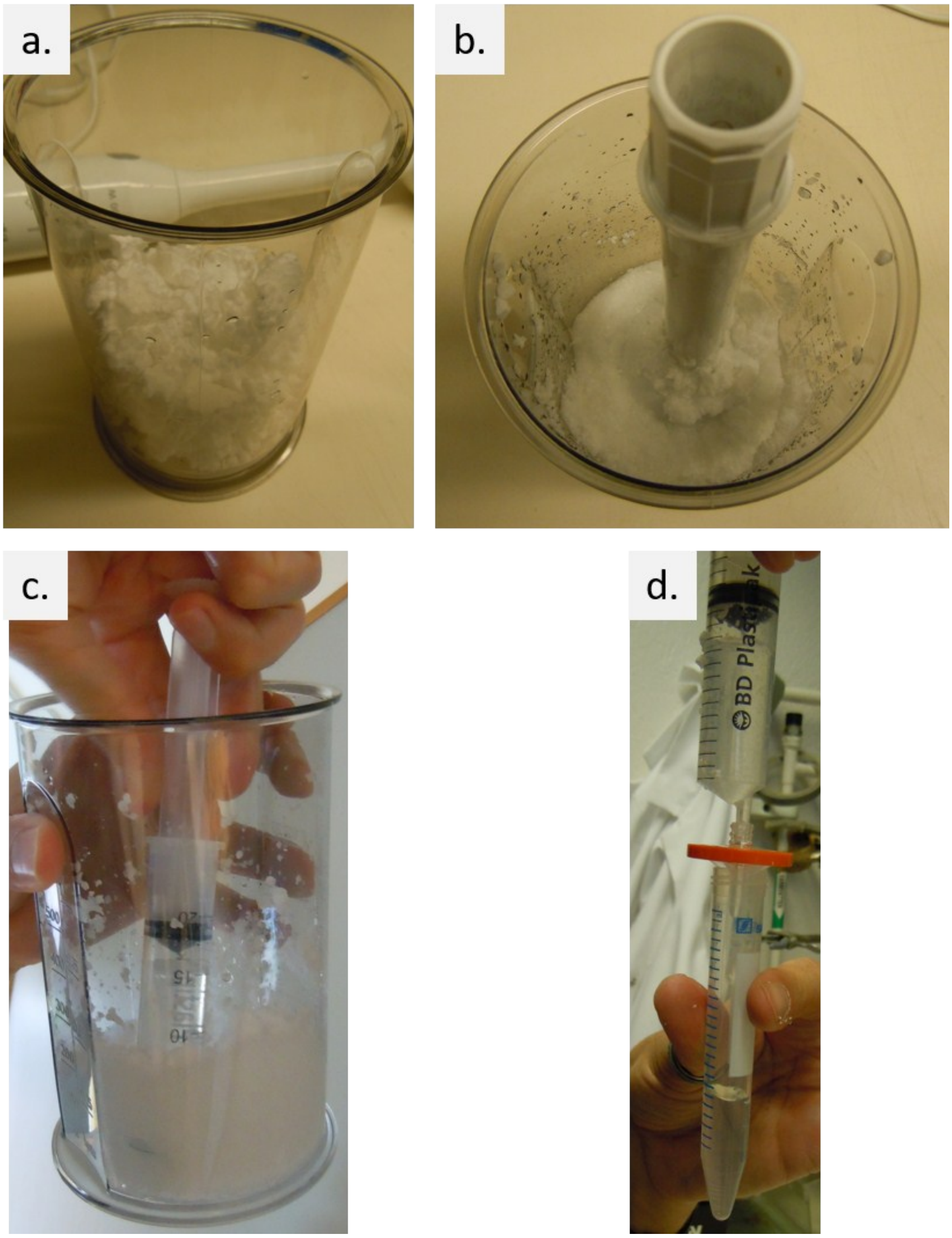

Figure S1. Showing different steps in the polystyrene nanoparticle manufacturing procedure. a. shows the beaker with the expanded polystyrene foam board broken into small pieces into the water. b. shows the "polystyrene smoothie" after 5 min treatment with the immersion blender. c. shows the withdrawal of the water part of the "polystyrene smoothie" with a syringe. d. shows the filtration of the "polystyrene smoothie".

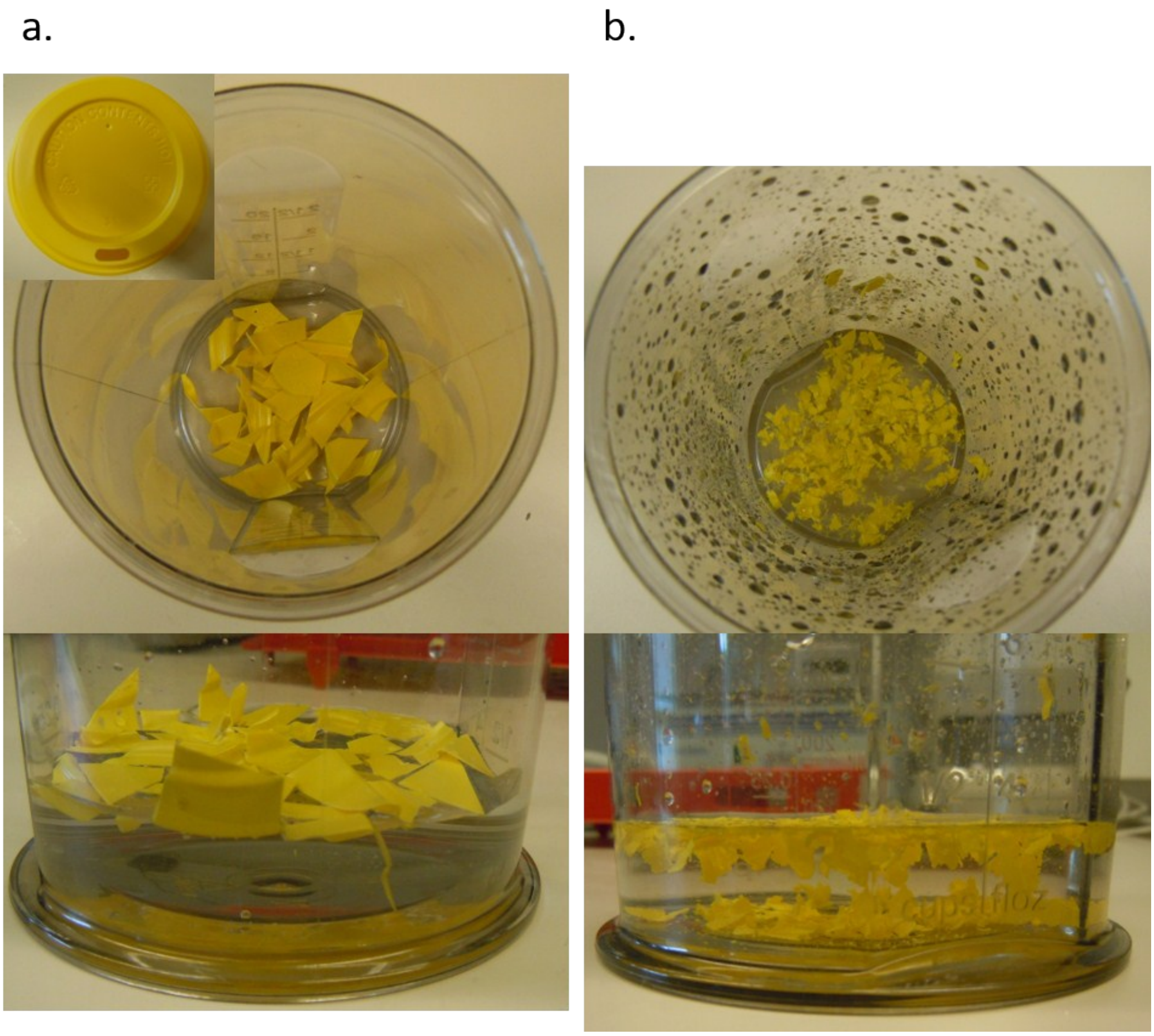

Figure S2. Production of particles using a coffee cup lid as starting material. a. showing the pieces in water from top and side view. Inset into the top view picture is a picture of the intact coffee lid. b. showing a top and side view of the mixture after 5 min treatment with the immersion blender.

Choice of immersion blender

Before reproducing the result shown in this article, make sure that your immersion blender does not produce nanoparticles by itself. We wanted a robust and efficient immersion blender to replace the one we used in this article and bought a professional kitchen blender (robot coupe Mini MP). However, we cannot use the new blender in our experiments since it by itself generates to high baseline concentration of nanoparticles of unspecific material.

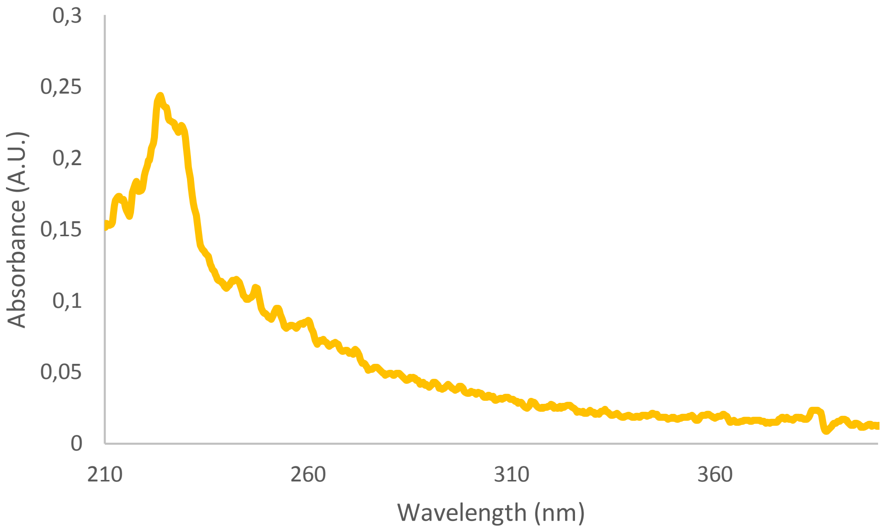

Figure S3. Absorption spectra for 80 nm carboxylated polystyrene particles from Bangs Laboratories Inc. USA.

Analytical HPLC

We loaded 5 different commercial polystyrene particles, from Bangs Laboratories Inc. USA, on the analytical HPLC column. The results are shown in figure S4.

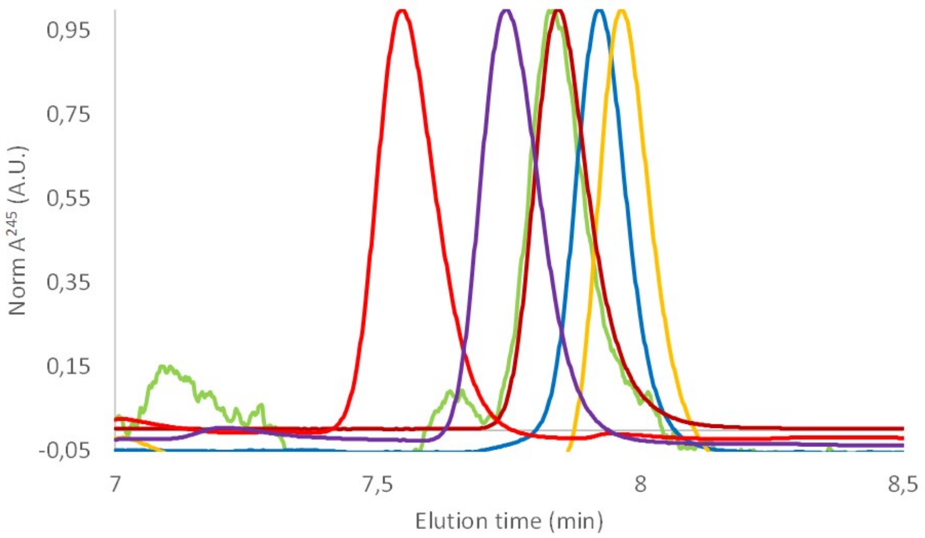

Figure S4. 50 nm positively charged PS in red, 60 nm negatively charged PS in blue, 50:50 mixture of 50 nm positively charged and 60 nm negatively charged PS in purple, 200 nm negatively charged PS in orange, 30 nm negatively charged PS in green, and 220 nm negatively charge PS in dark red.

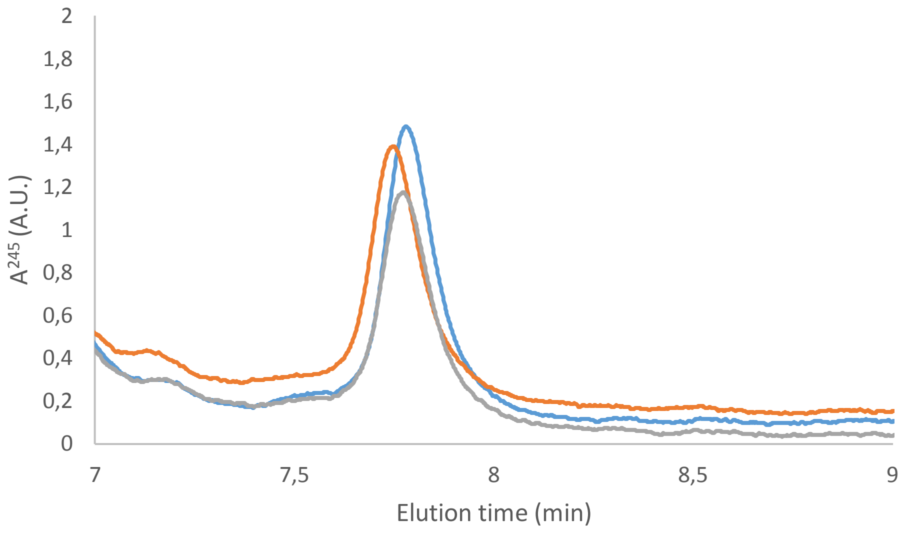

Figure S5. Three repeats of expanded foam samples showing the reproducibility of the elution time.

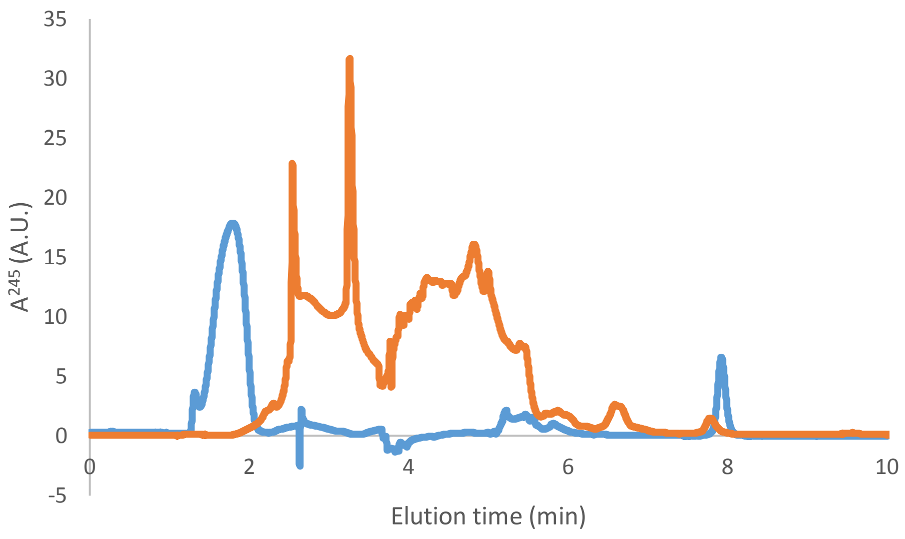

Figure S6. Showing an indication of purity of the samples. Bangs 60 nm PS-COOH particles, from Bangs Laboratories Inc. USA, in blue and the expanded foam board sample in orange.

NTA data

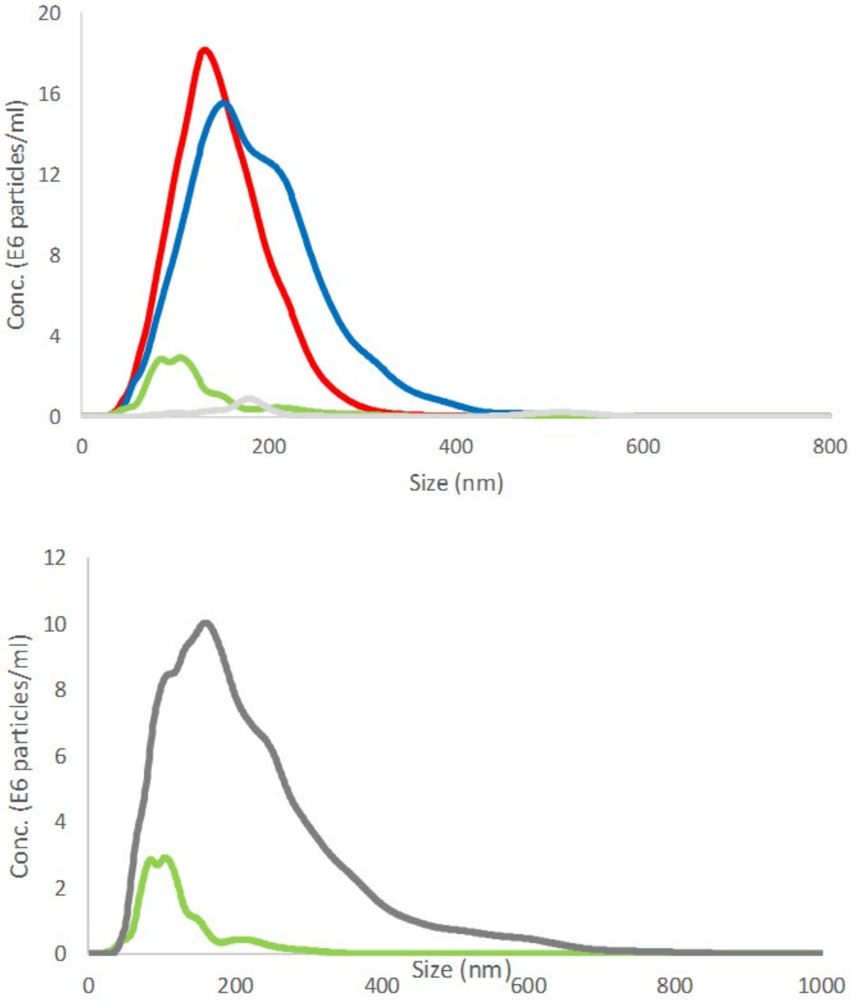

Figure S6. Example of the raw data from the NTA measurements. Top panel shows size distribution data for the expanded foam filter through 1.2 µm filter in blue, expanded foam filter through first 1.2 and then 0.45 µm filter in green, coffee lid sample in green, and control, just water mixed with the immersion blender and filter through 1.2 µm filter, in light grey. Lower panel shows the size distribution of the coffee lid sample before, green, and after, grey, concentration via lyophilisation.

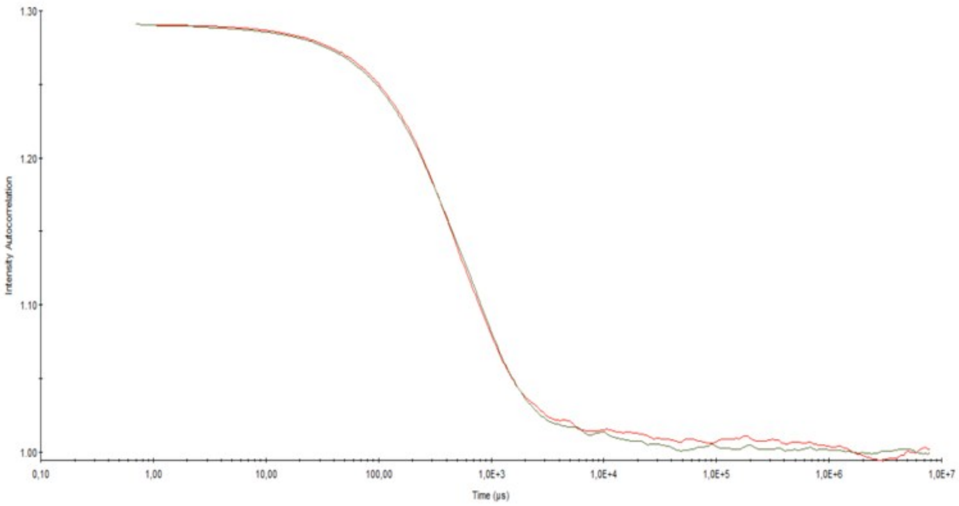

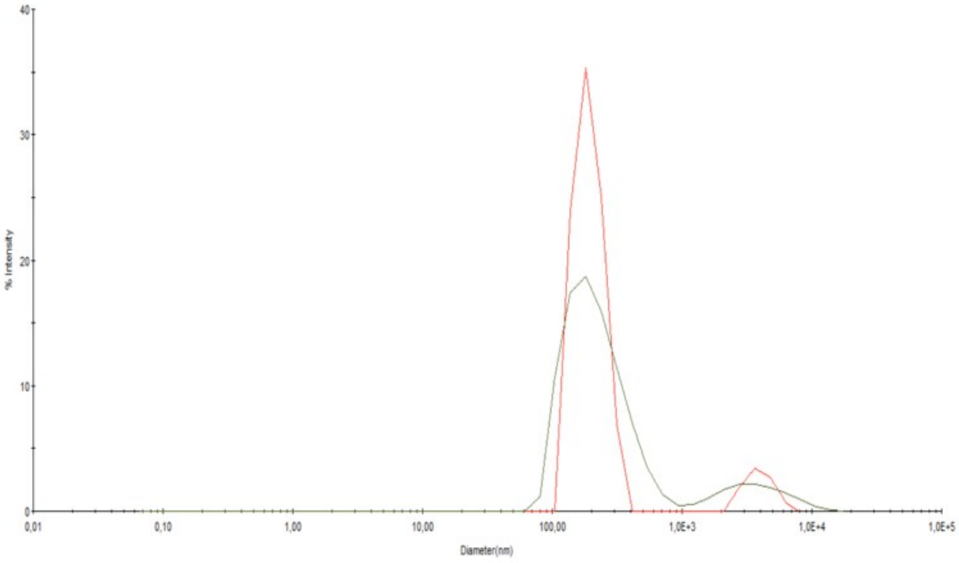

Figure S7. DLS data for expended foam sample filtered through 1.2 µm filter. Upper panel shows the correlation curves and the lower panel shows the corresponding size distribution.

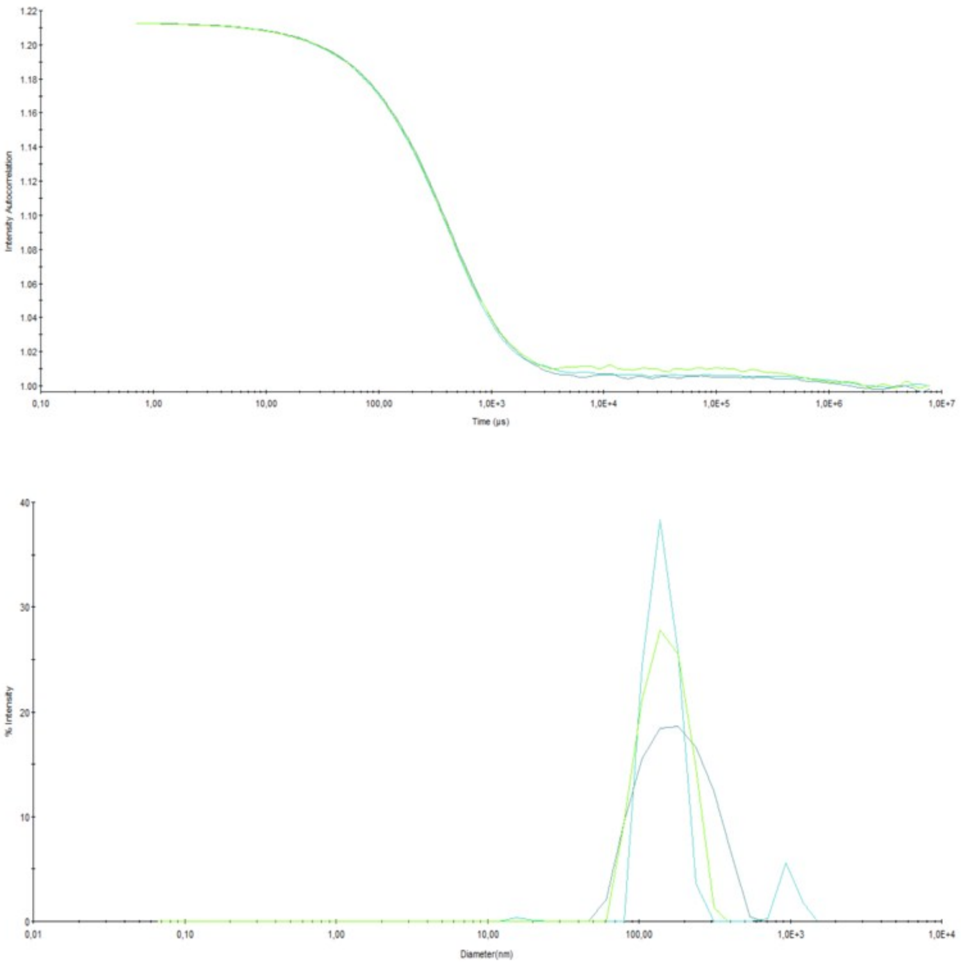

Figure S7. DLS data for expended foam sample filtered through 0.45 µm filter. Upper panel shows the correlation curves and the lower panel shows the corresponding size distribution.
